# Plant Defensive β-Glucosidases Resist Digestion and Sustain Activity in the Gut of a Lepidopteran Herbivore

**DOI:** 10.3389/fpls.2018.01389

**Published:** 2018-10-08

**Authors:** Daniel Giddings Vassão, Natalie Wielsch, Ana Maria de Melo Moreira Gomes, Steffi Gebauer-Jung, Yvonne Hupfer, Aleš Svatoš, Jonathan Gershenzon

**Affiliations:** ^1^Department of Biochemistry, Max Planck Institute for Chemical Ecology, Jena, Germany; ^2^Research Group Mass Spectrometry/Proteomics, Max Planck Institute for Chemical Ecology, Jena, Germany; ^3^Department of Entomology, Max Planck Institute for Chemical Ecology, Jena, Germany

**Keywords:** plant two-component defense, β-glucosidase, myrosinase, *Spodoptera*, frass proteomics, glucosinolate, benzoxazinoid, DIMBOA

## Abstract

Two-component activated chemical defenses are a major part of many plants’ strategies to disrupt herbivory. The activation step is often the β-glucosidase-catalyzed removal of a glucose moiety from a pro-toxin, leading to an unstable and toxic aglycone. While some β-glucosidases have been well studied, several aspects of their roles *in vivo*, such as their precise sites of enzymatic activity during and after ingestion, and the importance of particular isoforms in plant defense are still not fully understood. Here, plant defensive β-glucosidases from maize, white mustard and almonds were shown to resist digestion by larvae of the generalist lepidopteran *Spodoptera littoralis*, and the majority of the ingested activities toward both general and plant pro-toxic substrates was recovered in the frass. Among other proteins potentially involved in defense, we identified specific plant β-glucosidases and a maize β-glucosidase aggregating factor in frass from plant-fed insects using proteomic methods. We therefore found that, while *S. littoralis* larvae efficiently degraded bulk food protein during digestion, β-glucosidases were among a small number of plant defensive proteins that resist insect digestive proteolysis. These enzymes remain intact in the gut lumen and frass and can therefore further catalyze the activation of plant defenses after ingestion, especially in pH-neutral regions of the digestive system. As most of the ingested enzymatic activity persists in the frass, and only particular β-glucosidases were detected via proteomic analyses, our data support the involvement of specific isoforms (maize ZmGlu1 and *S. alba* MA1 myrosinase) in defense *in vivo*.

## Introduction

Many plants utilize activated plant chemical defenses to fend off herbivore attacks. These small molecular weight compounds are stored as biologically inactive pro-toxins in the intact tissue, and are then enzymatically activated to form bioactive toxic compounds after tissue damage by an attacking herbivore (Halkier and Gershenzon, 2006; Morant et al., 2008). This two-component system avoids the problem of auto-toxicity, as these defensive molecules can be stored without ill effects to the plants. A very common activation strategy is the enzymatic removal of a protecting glucose group by a β-glucosidase (Morant et al., 2008). The resulting aglycone is typically more reactive than the parent glucoside and accordingly displays increased toxicity. Furthermore, its lower polarity facilitates diffusion through cell membranes and penetration into the herbivore cells where toxicity is often exerted. In order to avoid uncontrolled and unnecessary activation, the two components of the defensive system (the glucosylated pro-toxin and the activating β-glucosidase) are kept spatially separated. These meet only when tissue integrity is lost, leading to mixing of the contents of different cells and cellular compartments. The increased water-solubility conferred by glycosylation allows for storage of millimolar concentrations of compounds such as glucosinolates, benzoxazinoids, and cyanogenic glucosides. While much is known about the chemistry and compartmentalization of activated defenses, it is not clear whether they are activated only on plant damage or also later in the herbivore gut.

**FIGURE 1:**
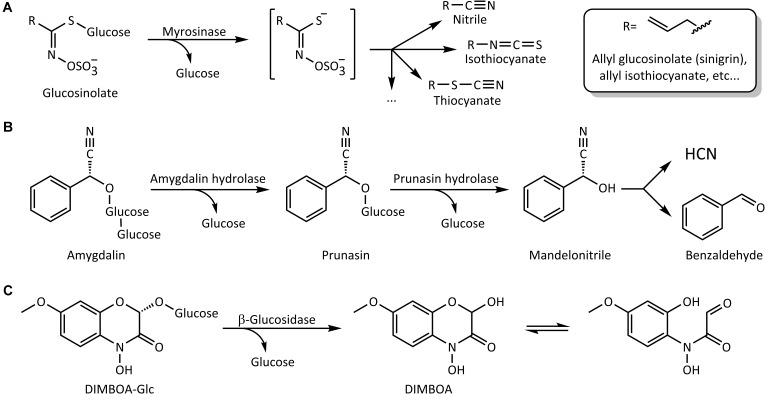
Schematic representation of some reactions catalyzed by plant β-glucosidases. **(A)** Activation of glucosinolates by myrosinase. **(B)** Step-wise hydrolysis of the cyanogenic diglucoside amygdalin. **(C)** Hydrolytic activation of the benzoxazinoid glucoside DIMBOA-Glc and formation of the open-ring aglycone form in solution.

Among activated plant chemical defenses, one of the best-studied systems involves glucosinolates and myrosinases that are mostly restricted to brassicaceous plants such as broccoli, mustard, and rapeseed (Halkier and Gershenzon, 2006). Hydrolysis of glucosinolates results in the liberation of glucose and an unstable *N*-(sulfooxy)alkylimidothioic acid aglycone that undergoes rearrangement in aqueous media to form compounds of varying toxicity. These products include isothiocyanates (mustard oils), nitriles, epithionitriles, and thiocyanates (**Figure 1A**) depending on the reaction conditions (e.g., pH, concentrations of metal cations and plant-derived specifier proteins) and the nature of the glucosinolate side chain (Bones and Rossiter, 2006; Wittstock and Burow, 2010; Jeschke et al., 2016b). Among those, isothiocyanates are considered particularly toxic, reacting with biological nucleophiles such as glutathione (GSH) and certain amino acid side-chains in proteins (Bruggeman et al., 1986; Mi et al., 2011; Mithöfer and Boland, 2012; Schramm et al., 2012; Jeschke et al., 2016a). The sugar moiety in glucosinolates is connected to the aglycone via an *S*-β-glucosidic bond. The β-glucosidases that activate glucosinolates are therefore β-thioglucoside glucohydrolases, commonly named myrosinases.

Cyanogenic glucosides are similarly activated by removal of their sugar group(s), leading to α-cyanohydrin aglycones. For example, one sugar molecule from the diglucoside amygdalin, found especially in fruits of the Rosaceae, is removed by amygdalin hydrolases to give prunasin, and this cyanogenic monoglucoside is further hydrolyzed into mandelonitrile and glucose by prunasin hydrolases (**Figure 1B**). The mandelonitrile aglycone thus formed may then dissociate into benzaldehyde and the very toxic hydrogen cyanide (HCN) non-enzymatically, especially under high pH (Fomunyam et al., 1985), or with enzymatic catalysis by mandelonitrile lyase (Swain and Poulton, 1994a,b). Emission of HCN, or cyanogenesis, occurs in a surprisingly large number of plant species of importance to humans, being a feature of grain crops such as oats, sorghum and maize, fruit crops such as apples and mangoes, and in the sub-Saharan staple food crop cassava.

Finally, benzoxazinoids (BXDs) are indole-derived defense compounds produced by a small number of agriculturally relevant grass (Poaceae) crops, including maize, wheat and rye (Frey et al., 1997; Niemeyer, 2009), and in scattered dicotyledonous species. They are mostly accumulated in intact plant tissues as the corresponding BXD glucosides. These lead, after glucose hydrolysis, to cyclic hemiacetal aglycones that equilibrate to α-oxoaldehydes by ring opening (**Figure 1C**) and are reactive toward a wide range of biological nucleophiles (Maresh et al., 2006; Dixon et al., 2012). The major BXD glucoside in young maize leaves is (2*R*)-2-β-D-glucopyranosyloxy-4-hydroxy-7-methoxy-2*H*-1,4-benzoxazin-3(4*H*)-one (DIMBOA-Glc), whose aglycone DIMBOA is considered to be one of the primary maize defensive chemicals against generalist leaf-chewing herbivores (Cambier et al., 2000; Glauser et al., 2011).

Glycosidases are a generally well-studied class of enzymes, due in part to their industrial utility. Plant β-glucosidases are classified as part of the Family 1 of glycoside hydrolases, catalyzing the hydrolysis of a terminal β-D-glucose residue via a mechanism in which the anomeric configuration of the glucose is retained. These β-glucosidases generally have a subunit molecular mass of 55–65 kDa, slightly acidic pH optima (pH 5–6), and an absolute specificity toward β-glucosides (Fowler, 1993). The tertiary structure of plant β-glucosidases belonging to family 1 glycoside hydrolases is highly conserved, as are the active site amino acids involved in binding of the sugar moiety. The residues that define the aglycone binding pocket are found at conserved positions in the active site, but are variable. The regions involved in oligomerization also vary among β-glucosidases. This is reflected in the diversity of quaternary structures observed in plant β-glucosidases, where the different active enzymes can be monomers, dimers, tetramers, or even higher oligomers. While the glucoside pro-toxins are typically stored in the vacuole, the subcellular localization of activating β-glucosidases differs in monocotyledonous and dicotyledonous plants. Monocotyledonous β-glucosidases contain an N-terminal transit peptide leading to localization to plastids. In dicotyledons, these activating enzymes contain an N-terminal signal that results in co-translational glycosylation and secretion (Morant et al., 2008). Protein glycosylation promotes stability and solubility and mediates protein localization and protein–protein interactions (Nastruzzi et al., 1996; Burmeister et al., 1997), but is not essential for full enzymatic activity (Zhou et al., 2012).

Myrosinases (E.C. 3.2.1.147), the thioglucosidases responsible for hydrolytic activation of glucosinolates, are commonly present in multigene families, with members possibly having different roles in different tissues. The *Arabidopsis thaliana* genome encodes for six myrosinase genes (*tgg*1-6), with two apparently being pseudogenes, and two genes being expressed each in aerial parts (*tgg*1/2) and underground tissues (*tgg*4/5) (Andersson et al., 2009; Zhou et al., 2012). More recently, additional *A. thaliana* proteins (PEN2 and PYK10) have been described to have myrosinase-like activities restricted toward indolic glucosinolates (Bednarek et al., 2009; Nakano et al., 2014), but their specific functions in plant defense against herbivores are still unclear.

The widely commercially available β-glucosidase from almonds, which produce cyanogenic glycosides, has been studied biochemically in great detail. While it was shown to artificially induce herbivory-like volatile emission in cabbages (Mattiacci et al., 1995), its potential roles *in planta* also remain to be better characterized. It can be isolated in large amounts from sweet almonds as a mixture of isozymes, and it has been massively used as a model enzyme in the study of β-glucosidase mechanism, kinetics, and chemical inactivation [for example (Shulman et al., 1976; Grover and Cushley, 1977; He and Withers, 1997)]. Its stability also allows its use in the synthesis of glucosides *in vitro* in high concentrations of organic solvents, exploiting the reversibility of the glucoside hydrolysis reactions [for example (Vic et al., 1995; Ducret et al., 2006)]. Nevertheless, while this enzyme accepts many compounds *in vitro*, information about whether it hydrolyzes cyanogenic glucosides *in vivo* is lacking.

In maize, BXD β-glucosidase reactions can be performed by two enzymes, ZmGlu1 and ZmGlu2 (Czjzek et al., 2000), which share 90% sequence identity. ZmGlu1 and ZmGlu2 hydrolyze a broad spectrum of artificial and natural compounds in addition to the benzoxazinoid DIMBOA-Glc (Czjzek et al., 2000, 2001). The maize β-glucosidase Zm-p60.1, of which ZmGlu1 is the corresponding allozyme, has been implicated in the release of active cytokinins from inactive stored cytokinin-*O*-glucosides (Campos et al., 1993; Zouhar et al., 1999; Zouhar et al., 2001).

Some maize proteins have been shown to interact directly with β-glucosidases resulting in the formation of large aggregates. Biochemical and immunological studies demonstrated that β-glucosidase activity could not be detected in zymograms of certain maize genotypes (so-called “nulls”) due to the enzyme occurring as large insoluble or poorly soluble quaternary complexes (Esen and Blanchard, 2000). This complexation is mediated by a β-glucosidase-aggregating factor (BGAF), a chimeric maize protein consisting of an N-terminal dirigent (disease response) domain and a C-terminal jacalin-related lectin domain (Kittur et al., 2007). An analogous jacalin-like lectin protein from wheat (*Triticum aestivum*), HFR1, has direct antifeedant effects and is involved in resistance against the Hessian fly (*Mayetiola destructor*) (Subramanyam et al., 2008), but its individual protein binding partners were not examined. The enzymatic activities of ZmGlu1 and ZmGlu2 are unaffected by BGAF binding, suggesting that interaction with BGAF does not change the conformation of the enzymes (Blanchard, 2001). Similar myrosinase-binding proteins (MBP) have been found in rapeseed (Falk et al., 1995) and in *A. thaliana* (Takechi et al., 1999; Takeda et al., 2008), where they also lead to formation of higher-molecular weight active complexes. However, whether these protein aggregates are stable in herbivore guts after ingestion and whether they play any roles in the hydrolysis of activated defenses *in vivo* is not yet known.

Nitrogen in the form of proteins and essential amino acids is considered the most limiting macronutrient in herbivorous diets, justifying the evolution of efficient and flexible cocktails of digestive proteases in lepidopteran herbivores (Terra and Ferreira, 1994; Kuwar et al., 2015). Accordingly, most bulk plant protein is effectively hydrolyzed after ingestion by these insects (Chen et al., 2005) and can be used for insect growth. However, a growing number of plant proteins have been described in insect frass, several of which remaining catalytically or biologically active after passing through the insect digestive tract and acting in plant defense both during and after ingestion (Chen et al., 2005, 2007; Chuang et al., 2013; Ray et al., 2016).

In studying plant two-component activated defenses, it is important to learn if plant β-glucosidases and other hydrolyzing enzymes critical to defense survive initial proteolysis and are active in the insect gut. Recent experiments using insects fed on maize leaves showed that maize β-glucosidases involved in benzoxazinoid metabolism are active even after going through insect digestion (Glauser et al., 2011; Wouters et al., 2014). Hence we have now examined the stability of plant defensive β-glucosidases after digestion by a generalist insect herbivore (*Spodoptera littoralis*) in order to better understand their roles in defense. We investigated three different β-glucosidases said to be involved in benzoxazinoid, glucosinolate, and cyanogenic glycoside activation, respectively, by determining their presence in protein extracts made from insect frass and comparing their catalytic activity in non-ingested material and after passage through the gut.

## Materials and Methods

### Materials

Experiments with maize (*Zea mays*) used seeds of the variety Badischer Gelber from Kiepenkerl (Bad Marienberg, Germany) while white mustard (*Sinapis alba*) seeds were obtained from N. L. Chrestensen (Erfurt, Germany). Maize and white mustard were sown in plastic pots (9 cm × 9 cm) with two plants per pot under controlled light and temperature conditions (16:8 h light/dark, day-time temperature 22°C, night-time 20°C). White mustard thioglucosidase (180 U/g toward sinigrin), almond (*Prunus dulcis*) β-glucosidase (5.2 U/mg toward salicin), sinigrin (allyl glucosinolate), amygdalin and *p*-nitrophenyl-β-D-glucoside (pNPG) were obtained from Sigma-Aldrich (Munich, Germany). Larvae of *S. littoralis* (Lepidoptera: Noctuidae) were reared from eggs kindly provided by Syngenta Crop Protection (Stein, Switzerland) on artificial diet modified from Bergomaz and Boppré (1986) at 18°C under 12:12 h light cycle, and used at the fourth instar. The diet was prepared as follows: 2.4 g agar-agar was boiled with 80 mL tap water and added to a mixture of 25 g white bean flour, 450 mg ascorbic acid, 250 mg 4-ethylbenzoic acid and 25 mL tap water. The diet was stirred until hand-warm (∼40°C) and then supplemented with 45 mg α-tocopherol in 625 μL canola oil and 200 μL 3.7% formaldehyde.

### Maize β-Glucosidase Extraction

About 100 maize kernels were washed with 80% ethanol for 2 min and 3% sodium hypochlorite with 0.5% Tween 20 for 15 min, then left in a Petri dish with water for 2 days to germinate, and sown as described above. Six- to seven-day-old seedlings were harvested, frozen and ground in liquid nitrogen and stored at -80°C prior to extraction. Enzyme extraction and cryoprecipitation were performed according to (Esen, 1992) with the final supernatant concentrated with Centricon Plus-70 regenerated cellulose membrane 30,000 NMWL ultracentrifugation filters (Merck Millipore, Hessen, Germany) for about 40 min. All operations were carried out between 0 and 4°C. The enzymatic activity of the extract was determined using the non-specific substrate pNPG as described below. The final enzymatic extract was adjusted to 1 U/mL.

### DIMBOA-Glc Extraction

About 70 maize kernels were germinated as above and grown in the dark. Seven-day-old seedlings were harvested, weighed, and quickly frozen and ground in liquid nitrogen. Plant material was extracted with 3 mL of methanol per gram of ground plant material, quickly mixing the ground material in the solvent to avoid degradation. The suspension was centrifuged at 3,400 *g* for 10 min and the supernatant was collected. The extraction and centrifugation steps were repeated two more times, and the combined supernatants were filtered. Ammonium sulfate was added to the filtrate and left overnight. The supernatant was then filtered and evaporated under vacuum, and the solution was lyophilized. After resuspension at 200 mg/mL in 0.5% aqueous formic acid (FA)/MeOH (1:1) the compound was fractionated and purified using semi-preparative HPLC. Purification of DIMBOA-Glc used a Supelcosil LC-18-DB semi-preparative column (25 cm × 10 mm I.D., 5 μm particle size, Sigma-Aldrich, maintained at 25°C) with a flow rate of 4 mL/min, using FA (0.005%) in water and acetonitrile as mobile phases A and B, respectively. The gradient was as follows: 10–20% B (10 min), 100% B (3 min), 10% B (3 min) with detection by UV absorption at 254 nm. The solvents where evaporated partially by using a rotary evaporator, while the remaining water was removed by lyophilization, with 37.2 mg of DIMBOA-Glc obtained as a white powder.

### Caterpillar Feeding and Collection of Diet and Frass Samples

**FIGURE 2:**
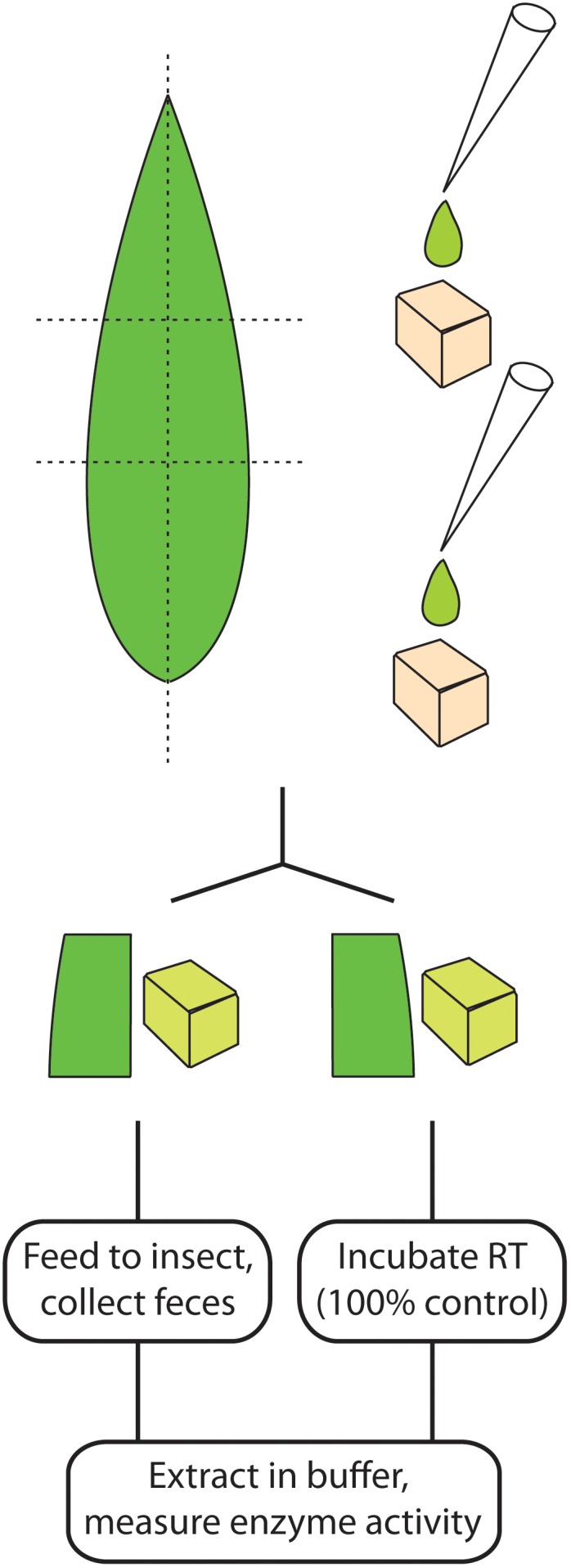
Schematic representation of enzyme feeding strategies from divided leaves (left) and in artificial diet cubes (right).

In artificial diet feeding experiments, *S. littoralis* caterpillars previously fed on artificial diet were switched to a cube of artificial diet (0.5 cm × 0.5 cm) to which pure enzyme (10 μL 0.026 U/mL *P. dulcis* β-glucosidase or 10 μL 10 U/mL *S. alba* myrosinase) or the semi-purified maize enzyme extract (10 μL 1 U/mL) was applied (**Figure 2**). Negative controls used 10 μL of the corresponding buffer. Each caterpillar was kept in an individual plastic cup and allowed to feed until the cube was consumed. An additional feeding experiment used bovine β-lactoglobulin (20 μL 10 mg/mL) added to the diet. Non-ingested samples used diet cubes with added enzyme kept under identical temperature conditions but in the absence of *S. littoralis* larvae. The resulting diet and frass samples were collected and extracted by adding two metal beads (diameter 3 mm) and 500 μL of the respective buffer used for each assay, followed by vigorous agitation with a paint shaker for 4 × 4 min, and centrifugation at 4,300 *g* at 4°C for 40 min. The supernatants were collected and assayed for enzymatic activity.

Feeding experiments with leaves instead of artificial diets were performed with maize and white mustard. For maize, one piece (about 3 cm × 1.5 cm) from a half of a leaf (divided longitudinally) from a 2-week-old plant was fed, with the other corresponding half frozen and used to determine non-ingested enzyme activity (**Figure 2**). For white mustard, five leaves from a 1-week-old plant were fed per caterpillar, with a set containing another five leaves used to quantify the initial myrosinase activity being fed. Six replicates were made for each feeding experiment. After complete ingestion of the glucosidase-containing diet, a small amount of control diet was offered to the insect, to allow for excretion of the material still present in the insect gut.

### Maize β-Glucosidase Activity Assay

In initial assays the non-specific β-glucosidase substrate *p*-nitrophenyl-β-D-glucopyranoside (pNPG) was used. For that, 500 μL of enzyme extract in 10 mM phosphate buffer at pH 7.1 were incubated with 500 μL 5 mM aqueous pNPG at 25°C for 5 min. The reaction was stopped by adding 1 mL 400 mM aqueous Na_2_CO_3_ and freezing in liquid nitrogen until further use, and the *p*-nitrophenol liberated was measured at 400 nm in a UV-Vis spectrophotometer shortly after thawing. Other enzyme assays used DIMBOA-Glc as substrate. The standard assay mixture for DIMBOA-Glc consisted of 5 μL enzyme extract (1 U/mL), 2 μL DIMBOA-Glc in DMSO (200 μM), and 43 μL 10 mM MOPS buffer pH 7.0. After incubation at 25°C for 20 min, 50 μL MeOH/FA (1:1) were added, and the samples were analyzed by LC-MS/MS as in (Wouters et al., 2014). Assays were performed in triplicate. Statistical testing for all enzyme activity results was performed with unpaired *t*-tests using the software GraphPad (La Jolla, CA, United States). β-Glucosidase activity was evident from both the decrease of the glucoside and concomitant appearance of the corresponding aglycone, which were quantified using external calibration curves.

### Almond β-Glucosidase Enzymatic Assay

The assay mixture consisted of 250 μL enzyme extract, 500 μL 0.1 M acetate buffer (pH 5.0) and 250 μL 20 mM aqueous pNPG. The reaction mixture was incubated at 37°C for 15 min and analyzed as above. Assays were performed in triplicate.

### *S. alba* Myrosinase Enzymatic Assay

The standard assay mixture consisted of 30 μL protein extract, 20 μL 50 mM aqueous sinigrin, and 200 μL of 50 mM citrate buffer (pH 5.5). After incubation for 60 min at 37°C, the reaction was stopped by addition of 25 μL glacial acetic acid and cooling on ice. The mixture was extracted with 250 μL CH_2_Cl_2_ containing 0.01% (v/v) hexyl-isothiocyanate as internal standard. A 100 μL aliquot of the CH_2_Cl_2_ fraction was removed and left overnight on 20 mg ammonium sulfate. Assays were performed in sextuplicate. The samples were analyzed by GC-MS, with enzymatic activities measured by quantification of released isothiocyanates using an external calibration curve of allyl-isothiocyanate.

### Analysis of Assay Products

LC-MS/MS analyses of DIMBOA-Glc β-glucosidase activity were performed as described in (Wouters et al., 2014). GC-MS analyses were performed in an Agilent 6890 series gas chromatograph (Agilent Technologies, Waldbronn, Germany) using an Agilent 19091S-433 capillary column (30 m × 0.25 mm × 0.25 μm), using 2 μL injection on splitless mode at 200°C. The temperature program was: 40°C for 3 min, a 10°C^∗^min^−1^ ramp to 130°C, a 60°C^∗^min^−1^ ramp to 300°C, held for 3 min. The total running time was 17.83 min.

### Spectrophotometry Analysis

A double-beam UV-Vis spectrophotometer (UV-2401PC, Shimadzu Corporation, Kyoto, Japan) equipped with integrating sphere was used for all spectrophotometry assays.

### Proteomic Analysis of Gel-Separated Proteins

Frass extracts for proteomic analyses were prepared as described above. Protein was quantified using the Bradford method, and about 200 μg of protein per sample were concentrated by lyophilization and separated using an Any kD Mini-Protean TGX Gel (Bio-Rad).

### In-Gel Digestion of Proteins

Protein bands between 20 and 120 kDa were cut out from the Coomassie-stained gels, cut into small pieces, washed several times with 25 mM NH_4_HCO_3_ and destained with 50% ACN/25 mM NH_4_HCO_3_. The proteins were then reduced in-gel with 10 mM dithiothreitol at 50°C for 1 h and alkylated with 55 mM iodoacetamide at room temperature in the dark for 45 min. Next, destained, washed, dehydrated gel pieces were rehydrated for 60 min in 0.5 μM solution of porcine trypsin (Promega) in 25 mM NH_4_HCO_3_ at 4°C and incubated overnight at 37°C. The tryptic peptides were extracted from gel pieces using 75% ACN/5% FA, and dried down in a SpeedVac. For LC-MS, analysis samples were reconstructed in 10 μL aqueous 1% FA (Shevchenko et al., 2007).

### Nano-UPLC-MS^*E*^ Analysis of Peptides

One to 8 μL of the peptide mixture was injected onto a nanoAcquity nanoUPLC system (Waters) online coupled to a Q-ToF HDMS mass spectrometer (Waters). Samples were initially transferred with 0.1% aqueous FA for desalting onto a Symmetry C18 trap-column (20 mm × 0.18 mm, 5 μm particle size) at a flow rate of 15 μL/min (0.1% aqueous FA), and peptides were subsequently eluted onto a nanoAcquity C18 analytical column (200 mm × 75 μm I.D., BEH 130 material, 1.7 μm particle size) at a flow rate of 350 nL/min with the following gradient: 1–30% B over 13 min, 30–50% B over 5 min, 50–95% B over 5 min, 95% B for 4 min, and a return to 1% B over 1 min (phases A and B composed of 0.1% aqueous FA and 100% acetonitrile containing 0.1% FA, respectively). The analytical column was re-equilibrated for 9 min prior to the next injection. LC-MS data were acquired in positive ESI mode under data-independent acquisition (MSE) controlled by MassLynx v4.1 software. The collision energy was set at 4 eV in low energy (MS) scans, and ramped from 15 to 40 eV in elevated energy (MSE) scans. The mass range (*m*/*z*) for both scans was 300–1900 and 50–1700 Da, respectively. The scan time was set at 1.5 s for both modes of acquisition with an inter-scan delay of 0.2 s. A reference compound, human glu-fibrinopeptide B [650 fmol/mL in 0.1% aqueous FA/acetonitrile (v/v, 1:1)], was infused continuously through a reference sprayer for external calibration.

### Data Processing and Protein Identification

ProteinLynx Global Server (PLGS) version 2.5.2 (Waters) was used for processing of raw files and for database searching. The continuum LC-MSE data were lock-mass corrected, smoothed, background subtracted, centered, deisotoped, and charge state reduced. Thresholds for low/high energy scan ions and peptide intensity were set at 150, 30, and 750 counts, respectively. Processed data were searched against the Swissprot database^1^ (downloaded on July 27, 2011) combined with protein sequences of *Z. mays*^2^ (downloaded on June 11, 2013) according to the described Ion Accounting Algorithm (Li et al., 2009). Database searches were performed at 4% FDR, using the following parameters: minimum number of product ion matches per peptide (5), minimum number of product ion matches per protein (7), minimum number of peptide matches (2), and maximum number of missed tryptic cleavage sites (1). Searches were restricted to tryptic peptides with a fixed carbamidomethyl modification for Cys residues, along with variable oxidation of Met. To avoid protein interference, identified hits were classified using a script based on the classification described for PAnalyzer software (Prieto et al., 2012). According to this algorithm, proteins were divided into four groups: conclusive, non-conclusive, indistinguishable, and ambiguous. Only proteins classified as conclusive hits (proteins with at least one unique peptide) were considered as confident matches.

### Estimation of Gene Expression Levels

Gene expression was estimated using the maize eFP browser of the Bio-Analytic Resource at the University of Toronto^3^ using the Sekhon et al. atlas setting (Winter et al., 2007; Sekhon et al., 2011) referring to data from B73 maize. Absolute expression values in the topmost leaf at growth stage V3 [“V3_Topmost leaf” (Sekhon et al., 2011)] are reported.

## Results

Plants produce β-glucosidases that activate anti-herbivore defenses by cleaving various β-glucoside pro-toxins. In order to analyze the stability of these β-glucosidases during digestion by larvae from a generalist lepidopteran pest, we fed known quantities of these enzymes to *S. littoralis* caterpillars (**Figure 2**) and quantified the enzymatic activities in non-ingested material and post-ingestion. Initial *in vitro* β-glucosidase assays using the general substrate *p*-nitrophenyl-β-D-glucoside (pNPG) revealed that non-specific β-glucosidases are present in frass from untreated larvae that arise from their diet and from the insect’s own endogenous activities, but these enzymes were not capable of cleaving specific plant defense compounds [such as DIMBOA-Glc (Glauser et al., 2011)]. Therefore, when using pNPG, the non-specific activities were measured (and corresponded to 5–30% of the added enzyme activities, depending on the enzyme being combined) and subtracted from activities measured in samples containing the added β-glucosidases.

### Maize β-Glucosidase (Involved in Benzoxazinoid Hydrolysis) Was Active After Insect Digestion

**FIGURE 3:**
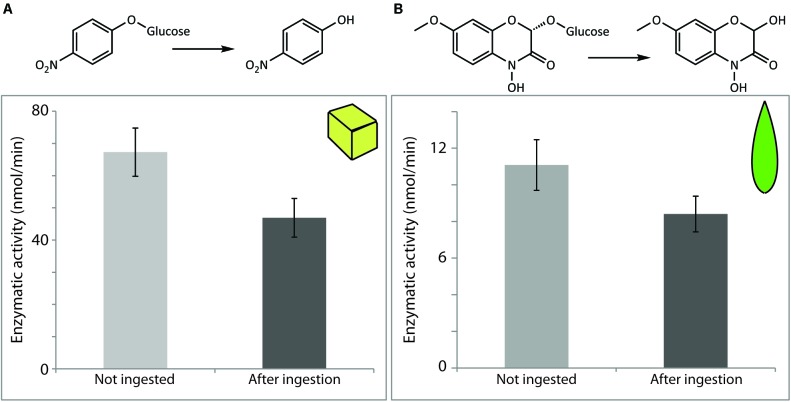
Maize β-glucosidases were recovered in active form after digestion by *S. littoralis* larvae. Enzymatic activity levels were determined in non-ingested material and after digestion of diet cubes spiked with semi-purified maize β-glucosidase **(A)**, or after feeding on maize leaf tissue **(B)**. Activities were measured either using the non-specific β-glucosidase substrate pNPG **(A)** or the endogenous plant substrate DIMBOA-Glc **(B)**. Shown are the means ± standard errors [*N* = 3 in both analyses; *P* = 0.2 **(A)** and *P* = 0.3 **(B)**].

Previous studies had shown that maize BXD-Glc β-glucosidases are active throughout the insect gut (Glauser et al., 2011), including within the hindgut and frass (Wouters et al., 2014). In order to quantify these observations, we first fed caterpillars on an artificial diet to which we applied a maize β-glucosidase enzyme extract in a similar amount/concentration to that in leaves. The resulting frass was then collected and extracted. Assays using pNPG indicated that maize β-glucosidases added to the diet were indeed excreted in active form, with 69.6% of the initial activity fed to the larvae being detected in the frass extracts (**Figure 3A**).

To assess how other plant-derived proteins might influence the digestion of these maize defensive β-glucosidases, caterpillars were subsequently fed a controlled amount of young maize leaf tissue. Leaves were divided along the mid-vein, and the matching leaf pieces (i.e., from the opposing sides of the leaf blade, see **Figure 2**) were (a) fed to larvae and (b) used to quantify the initial β-glucosidase activity being fed. Quantification of activity was done with DIMBOA-Glc, a more specific and biologically relevant substrate than pNPG. We recovered 75.9% of the enzyme activity initially fed to the larvae, further confirming that the maize BXD β-glucosidases maintain most of their activity throughout the caterpillar gut (**Figure 3B**).

### Myrosinase (Involved in Glucosinolate Hydrolysis) Was Active After Insect Digestion

Similarly, we quantified the activity of *S. alba* myrosinase remaining after digestion by *S. littoralis* larvae, using pure enzyme added to an artificial diet and by feeding with *S. alba* leaves. In both cases, enzymatic activities in non-ingested material and post-digestion were measured using a specific myrosinase substrate, sinigrin (allyl glucosinolate), with quantification of its main hydrolysis end-product, allyl isothiocyanate.

**FIGURE 4:**
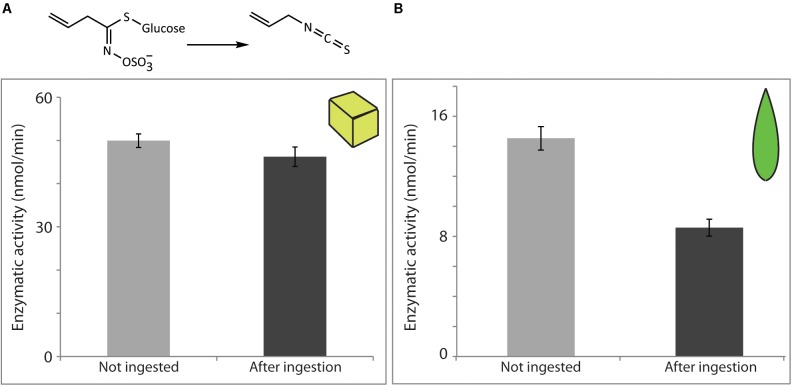
*Sinapis alba* myrosinase activity was resistant to digestion by *S. littoralis* larvae and detected in larval frass. Enzymatic activity levels were determined in non-ingested material and after digestion of diet cubes spiked with pure enzyme **(A)** or of *S. alba* leaf material **(B)**. Activities were measured using the myrosinase-specific substrate sinigrin. Shown are the means ± standard errors [*N* = 6 in both analyses; *P* = 0.2 **(A)** and *P* < 0.0001 **(B)**].

Myrosinase was also recovered in active form after digestion by the larvae. When the pure commercial enzyme was fed in artificial diet, 92.6% of the myrosinase activity administered was successfully recovered in the insect feces (**Figure 4A**), while 59.1% was recovered when comparing the activity in frass to that present in the corresponding unfed leaf (**Figure 4B**). The potential effects of the presence of ascorbate on the measured myrosinase activities were not tested, but this compound is known to increase *S. alba* myrosinase activity (Pihakaski and Pihakaski, 1978). Variation in its concentration between non-ingested material and post-digestion samples from plants and artificial diets might therefore account for some of the contrast in the apparent recovered activities.

### Almond β-Glucosidase Was Active After Insect Digestion

**FIGURE 5:**
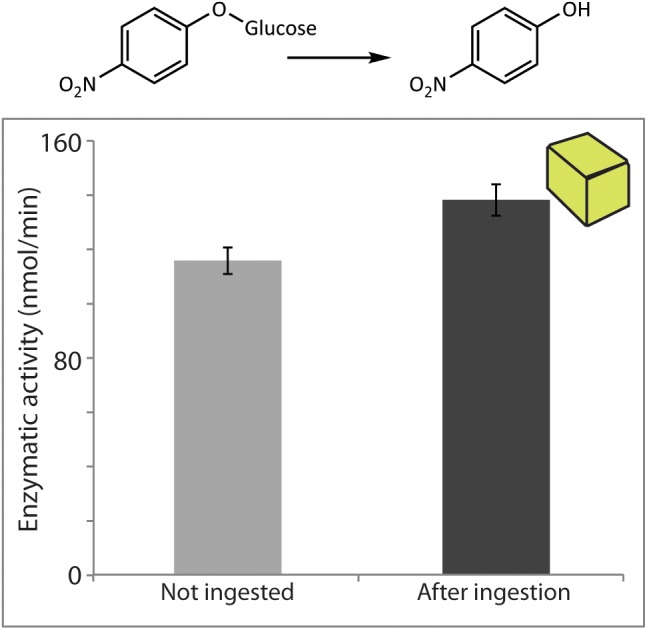
*Prunus dulcis* glucosidase activity was resistant to digestion by *S. littoralis* larvae and detected in larval frass. Enzymatic activity levels were determined in non-ingested material and after digestion of diet cubes spiked with pure enzyme. Activities were measured using the general β-glucosidase substrate pNPG. Shown are the means ± standard errors (*N* = 3; *P* = 0.09).

For quantification of the enzymatic activity of *P. dulcis* β-glucosidase after digestion by *S. littoralis*, pure commercial enzyme added to an artificial diet was used for the feeding experiment. Enzymatic assays using amygdalin did not lead to detectable amounts of its expected hydrolysis products. Enzymatic assays performed with the non-specific substrate pNPG, on the other hand, indicated that activity was quantitatively recovered after ingestion by the larvae (**Figure 5**). The measured post-digestion activities were slightly higher than those for the undigested control, but the differences were not statistically significant (*p* = 0.09) and are likely due to natural variation in levels of endogenous insect β-glucosidase activities.

### Proteomic Analyses

Protein extracts for proteomic analyses were prepared from maize and white mustard leaves, both in non-ingested material and post-ingestion. After separation by SDS–PAGE, gel regions were excised and digested with trypsin. The resulting peptides were analyzed by LC-MS using data-independent acquisition (DIA). In this approach, referred to as MS^E^, data were comprehensively acquired with an alternating mode of acquisition: a low energy (MS) mode that provided information on the mass of eluted intact peptide ions (precursors), and an elevated energy mode (MS^E^) when fragmentation of these ions occurred. The ion accounting algorithm implemented in the software PLGS (Waters) was applied for searching parallel fragmentation of multiple precursor ions, matching fragment ions to their corresponding precursor peptides while taking into account their chromatographic coelution in LC-MS/MS.

**FIGURE 6:**
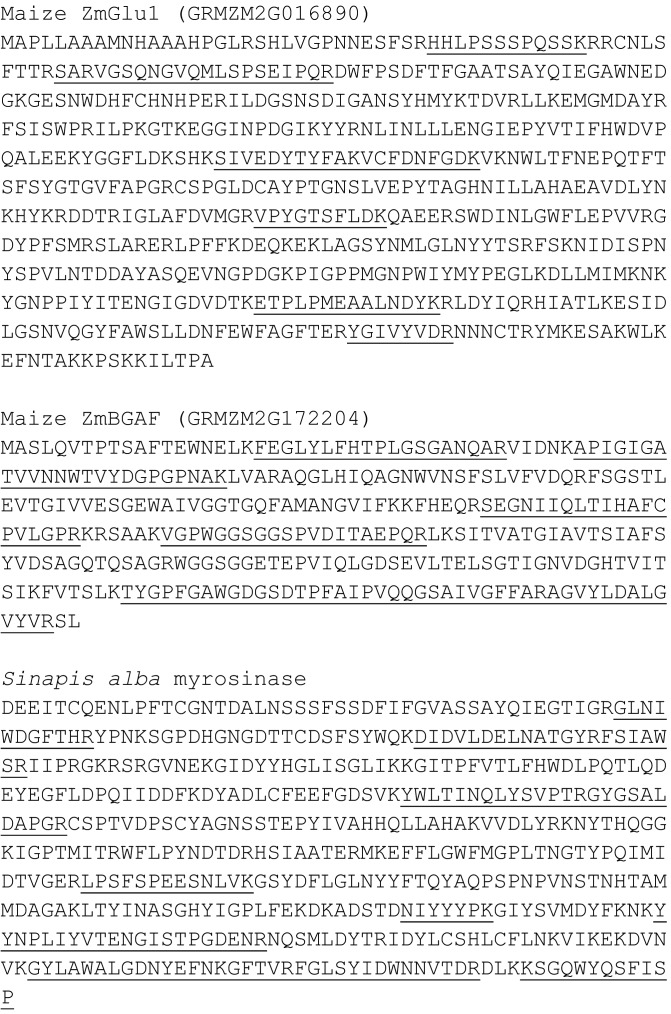
LC-MS^E^-based proteomic identification of peptides (marked with an underline) derived from maize β-glucosidase ZmGlu1, maize β-glucosidase aggregating factor (BGAF), and *S. alba* myrosinase in frass samples.

These analyses led, in the case of maize, to detection of six BXD β-glucosidase-derived peptides in frass material (**Figure 6**). Two maize β-glucosidases have been identified to act on DIMBOA-Glc (ZmGlu1 and 2), but we were only able to confidently identify peptides derived from ZmGlu1. Seven peptides derived from the maize β-glucosidase aggregating factor (BGAF) protein were observed in frass extracts. Eleven peptides derived from *S. alba* myrosinase were found in frass extract samples, with unambiguous peptides indicating that the enzyme detected here is the isoform MA1. In contrast to the peptides belonging to the two maize proteins, the peptides corresponding to myrosinase were spread over a broad region of the SDS–PAGE gel (data not shown). This could result from partial deglycosylation/digestion of the proteins during passage through the gut.

Peptides belonging to 13 additional maize proteins were also detected in *S. littoralis* frass extracts (**Supplementary Table S1**), including some with predicted functions matching those previously observed in the frass of maize-fed *Spodoptera frugiperda* larvae (Chuang et al., 2013). Expression levels of the corresponding genes in maize leaves (Sekhon et al., 2011) are scattered widely (**Supplementary Table S1**) and do not correlate with numbers of detected peptides or sequence coverage, supporting a differential resistance to insect proteolysis among plant proteins. Many differences were observed in the general profiles of plant-derived polypeptides in total leaf extracts (**Supplementary Table S2**) relative to those isolated from frass (**Supplementary Table S1**). One of the major distinctions was in the large subunit (rbcL) of Rubisco (UniProt P00874) considered a marker for bulk leaf protein (Chen et al., 2005, 2007). In contrast to the good (48%) coverage of rbcL primary sequence in leaf extracts, our proteomic analyses did not detect any peptides derived from rbcL in frass extracts, supporting the degradation of bulk leaf protein during passage through the larval gut. In order to confirm this finding, we fed diet cubes containing bovine β-lactoglobulin in similar concentrations to those above for β-glucosidases, and analyzed non-ingested material and post-digestion samples. We could not detect a band corresponding to β-lactoglobulin with SDS–PAGE and Coomassie staining in insect-digested samples, and no peptides matching this protein could be found in frass proteomic data within the size range analyzed (∼7–35 kDa), again indicating that efficient proteolysis of bulk protein takes place during digestion by this herbivore.

## Discussion

Previous work had identified defensive plant proteins in insect frass following passage through the insect digestive system (Chen et al., 2005, 2007; Chuang et al., 2013; Ray et al., 2016), but their biological activities were assessed only in a few specific cases. Here, we fed three β-glucosidases presumed to be involved in activating plant defenses to *S. littoralis* caterpillars and found that they remained active after passage through the gut. A maize β-glucosidase involved in benzoxazinoid hydrolysis, a *S. alba* myrosinase involved in glucosinolate activation, and an almond β-glucosidase associated with cyanogenic glycoside accumulation were all detected in frass using both *in vitro* enzymatic assays and proteomic analyses. Our proteomic analyses identified several additional proteins in extracts from frass of maize-fed larvae that could participate in plant defense, such as oxidases, proteases, and other glycoside hydrolases. Interestingly, both arginine decarboxylase and agmatine deiminase were observed, suggesting that these may function sequentially to deplete the essential amino acid arginine from the food bolus as part of a defensive strategy. Other predicted glycosyl hydrolases were also detected, and their utilization of benzoxazinoids as substrates and roles in defense remain to be explored.

The persistence of these proteins and corresponding activities in the frass means they are at least partially resistant to the proteases, high pH (9.5–11) and detergents present in *S. littoralis* guts. These results are in agreement with previous reports showing that other β-glucosidases, such as cassava cyanogenic β-glucosidase isozymes (linamarases) and maize and almond β-glucosidases are stable against short treatments with trypsin, high temperature and chemical denaturants (Mkpong et al., 1990; Esen and Gungor, 1993). This unusual resistance may be a general trait of plant β-glucosidases and is an important aspect of their commercial value as industrial biocatalysts. In contrast to β-glucosidases which remained active in the resulting frass, bulk dietary proteins like Rubisco and artificially added bovine β-lactoglobulin were efficiently degraded in the *S. littoralis* gut.

The biophysical proprieties that contribute to the high stability of β-glucosidases remain to be fully understood, but some structural features common among them may be responsible. Alignment and structural superimposition have revealed that, despite relatively low primary-structure similarities (17–44%), the tertiary structures of family 1 β-glucosidases are remarkably similar. Several β-glucosidases, including those studied here, have a tight and stable folded core, presumably enabling activity over a wide range of conditions. Some amino acids present in the binding and catalytic sites of these glucosidases, e.g., the peptide motifs TFNEP and ITENG, are also highly conserved and could help stabilize the protein core. In contrast to the tertiary structures, a diversity of β-glucosidase quaternary structures has been observed; oligomerization may lead to variable functions or allow specific regulation of bioactivation of defense compounds.

Enzyme stability might also be influenced by glycosylation. We detected two myrosinase-derived peptides (those containing N90 and N482) that contain residues proposed (Burmeister et al., 1997) to be modified by sugars. Their presence in our frass extracts coupled with the relatively broad migration of the parent protein in SDS–PAGE suggest that these proteins which are glycosylated may have been partially cleaved and deglycosylated during digestion. The actual contribution of these modifications to protein activity is still unclear, as previous studies have demonstrated that myrosinase glycosylation is not essential for activity (Zhou et al., 2012), while monocotyledonous glucosidases are not glycoproteins. However, glycosylation may contribute to stability with deglycosylated proteins being more susceptible to further attack by insect gut proteases, oxidants, electrophiles (including their enzymatic products), and to variation in temperature and pH.

Further features responsible for protein stability in insect guts have been revealed by investigations of maize ZmGlu1. The dimerization of this protein is stabilized by a disulfide bridge that shields a cluster of hydrophobic residues in the active site from the solvent (Rotrekl et al., 1999; Zouhar et al., 2001). ZmGlu1 also has a high number of proline residues as well as several intramolecular ion pairs and hydrogen and electrostatic bonds (Czjzek et al., 2001), which give the protein thermal stability and resistance to denaturing agents. The average number of hydrogen bonds per residue is rather high (>1) in ZmGlu1 and *S. alba* myrosinase MA1 (Czjzek et al., 2001).

Our proteomic analyses also support the assignment of more specific *in vivo* roles to individual β-glucosidase isoforms in spite of their overlapping substrate preferences *in vitro*. For example, ZmGlu1 but not ZmGlu2, and *S. alba* MA1 but not the other annotated *S. alba* myrosinases, were unambiguously identified in insect frass extracts. As most of the catalytic activity of the ingested foliage was still present in these frass extracts, these particular enzymes may therefore be the isoforms most relevant in activating their respective plant defensive substrates. However, this is in contrast to a previous proteomic analysis of frass from maize-fed *S. frugiperda* larvae, where ZmGlu2 was abundantly detected (Ray et al., 2016). Future studies are needed to clarify the comparative stabilities and substrate preferences of these close homologs and dissect their functions. The degraded isoforms may fulfill other roles, such as hydrolysis of cytokinins by ZmGlu enzymes (Brzobohatý et al., 1993). The almond enzyme, on the other hand, was found not to hydrolyze the cyanogenic diglucoside amygdalin *in vitro*, but may still act to hydrolyze prunasin (the corresponding monoglucoside), although this possibility was not examined and its activity was measured using the general glucosidase substrate pNPG.

The maize β-glucosidase aggregating factor (BGAF) whose aggregation of ZmGlu might protect the latter from insect proteases (Kittur et al., 2007) also resists the digestive system of *S. littoralis*. However, the roles of such aggregation factors have not been fully determined. Our recovery of maize BXD β-glucosidase activity did not differ between frass derived from feeding whole plant tissues (containing BGAF) and that from feeding semi-purified β-glucosidase preparations (presumably without BGAF), suggesting that BGAF did not protect against digestive inactivation. However, strongly interacting protein partners could have remained bound to ZmGlu during our simple purification procedure.

Insect gut pH can influence enzyme stability and also have a direct impact on catalytic activity. While larval Orthoptera, Hemiptera, and the larvae of most coleopteran families have slightly acidic to neutral midguts, many larvae of Lepidoptera, Diptera, and scarab beetles (Coleoptera) have highly alkaline midguts (Terra and Ferreira, 1994). In previous work, we observed that the midgut lumen of *S. frugiperda* fed on maize leaves is alkaline, but becomes neutral toward the hindgut (Wouters et al., 2014). While maize DIMBOA-Glc β-glucosidases were active under both neutral and basic conditions, their enzymatic activity was much higher at pH 7.0 (closer to their slightly acidic pH optima) than at pH 10.0 (Wouters et al., 2014). This resulted in slow hydrolysis of DIMBOA-Glc in the alkaline fore- and midgut, but extensive activation in the hindgut and neutral rectum where absorption of water and salts takes place. In the case of *Zygaena filipendula*, the cyanogenic β-glucosidases of *Lotus* spp. also had lower activity in the highly alkaline gut significantly reducing cyanogenesis (Pentzold et al., 2014). Therefore, by lowering the activity of these β-glucosidases, the high pH observed in the midgut (but not hindgut) lumen of these and other lepidopteran herbivores may help the insect to partially counteract the resistance of these defensive β-glucosidases to proteolytic inactivation in the gut.

## Conclusion

Here, we have shown that selected plant β-glucosidases catalyzing the activation of chemical defenses survive digestion in a generalist lepidopteran herbivore, and are detectable in frass using *in vitro* activity assays and proteomic analyses. Although the structural features responsible for persistence in the digestive tract remain to be fully demonstrated, these enzymes seem particularly resistant because of their compact tertiary structure and the glycosylation of several residues, which have optimized them for resisting the protease-rich and high pH gut of lepidopteran herbivores. In parallel these features have allowed the use of such plant β-glucosidases in industrial applications even in the presence of detergents and organic solvents. The resistance to insect gut proteases even under high pH is presumably a result of natural selection on these enzymes to catalyze the continuous release of toxic aglycones in the herbivore gut, especially in the hindgut and rectum where the lumen reverts to more neutral conditions. Further investigations are needed to establish this extended role of β-glucosidases in the herbivore gut, and to determine which features of the proteins or their binding partners are responsible.

## Author Contributions

DV and NW designed the experiments. DV, NW, AG, SG-J, and YH acquired and analyzed the data. DV, NW, AG, AS, and JG interpreted the data and drafted the article.

## Conflict of Interest Statement

The authors declare that the research was conducted in the absence of any commercial or financial relationships that could be construed as a potential conflict of interest.

## Footnotes

1 http://www.uniprot.org/

2 http://www.phytozome.net/

3 http://bar.utoronto.ca/efp_maize

## Supplementary Material

TABLE S1 Maize proteins detected in *S. littoralis* frass extracts and their predicted functions, number of peptides observed by LC-MS and corresponding sequence coverage, and leaf expression levels (B73 maize).

TABLE S2 Proteins detected during LC-MS proteomic analysis of maize leaf extracts.

## References

[B1] Andersson D. Chakrabarty R. Bejai S. Zhang J. Rask L. Meijer J. (2009). Myrosinases from root and leaves of *Arabidopsis thaliana* have different catalytic properties. *Phytochemistry* 70 1345–1354. 10.1016/j.phytochem.2009.07.036 19703694

[B2] Bednarek P. Piślewska-Bednarek M. Svatoš A. Schneider B. Doubský J. Mansurova M. (2009). A glucosinolate metabolism pathway in living plant cells mediates broad-spectrum antifungal defense. *Science* 323 101–106. 10.1126/science.1163732 19095900

[B3] Bergomaz R. Boppré M. (1986). A simple instant diet for rearing Arctiidae and other moths. *J Lepid Soc.* 40 131–137.

[B4] Blanchard D. J. (2001). Identification of β-glucosidase aggregating factor (BGAF) and mapping of BGAF binding regions on maize β-glucosidase. *J Biol. Chem.* 276 11895–11901. 10.1074/jbc.M008872200 11096099

[B5] Bones A. Rossiter J. (2006). The enzymic and chemically induced decomposition of glucosinolates. *Phytochemistry* 67 1053–1067. 10.1016/j.phytochem.2006.02.024 16624350

[B6] Bruggeman I. M. Temmink J. van Bladeren P. J. (1986). Glutathione-and cysteine-mediated cytotoxicity of allyl and benzyl isothiocyanate. *Toxicol. Appl. Pharmacol.* 83 349–359. 10.1016/0041-008X(86)90312-1 3961819

[B7] Brzobohatý B. Moore I. Kristoffersen P. Bako L. Campos N. Schell J. (1993). Release of active cytokinin by a beta-glucosidase localized to the maize root meristem. *Science* 262 1051–1054. 10.1126/science.8235622 8235622

[B8] Burmeister W. P. Cottaz S. Driguez H. Iori R. Palmieri S. Henrissat B. (1997). The crystal structures of *Sinapis alba* myrosinase and a covalent glycosyl–enzyme intermediate provide insights into the substrate recognition and active-site machinery of an S-glycosidase. *Structure* 5 663–676. 10.1016/S0969-2126(97)00221-9 9195886

[B9] Cambier V. Hance T. de Hoffmann E. (2000). Variation of DIMBOA and related compounds content in relation to the age and plant organ in maize. *Phytochemistry* 53 223–229. 10.1016/S0031-9422(99)00498-7 10680175

[B10] Campos N. Bako L. Brzobohatý B. Feldwisch J. Zettl R. Boland W. (1993). “Identification and characterization of a novel phytohormone conjugate specific β-glucosidase activity from maize,” in *Proceedings of the ACS Symposium Series. Biochemistry and Molecular Biology* Vol. 533 ed. Esen A. (Washington, DC: American Chemical Society), 205–213. 10.1021/bk-1993-0533.ch014

[B11] Chen H. Gonzales-Vigil E. Wilkerson C. G. Howe G. A. (2007). Stability of plant defense proteins in the gut of insect herbivores. *Plant Physiol.* 143 1954–1967. 10.1104/pp.107.095588 17416643 PMC1851804

[B12] Chen H. Wilkerson C. G. Kuchar J. A. Phinney B. S. Howe G. A. (2005). Jasmonate-inducible plant enzymes degrade essential amino acids in the herbivore midgut. *Proc. Natl. Acad. Sci. U.S.A.* 102 19237–19242. 10.1073/pnas.0509026102 16357201 PMC1323180

[B13] Chuang W.-P. Herde M. Ray S. Castano-Duque L. Howe G. A. Luthe D. S. (2013). Caterpillar attack triggers accumulation of the toxic maize protein RIP2. *New Phytol.* 201 928–939. 10.1111/nph.12581 24304477

[B14] Czjzek M. Cicek M. Zamboni V. Bevan D. R. Henrissat B. Esen A. (2000). The mechanism of substrate (aglycone) specificity in β-glucosidases is revealed by crystal structures of mutant maize β-glucosidase-DIMBOA, -DIMBOAGlc, and -dhurrin complexes. *Proc. Natl. Acad. Sci. U.S.A.* 97 13555–13560. 10.1073/pnas.97.25.13555 11106394 PMC17614

[B15] Czjzek M. Cicek M. Zamboni V. Burmeister W. P. Bevan D. R. Henrissat B. (2001). Crystal structure of a monocotyledon (maize ZMGlu1) β-glucosidase and a model of its complex with p-nitrophenyl β-D-thioglucoside. *Biochem J.* 354 37–46. 10.1042/bj3540037 11171077 PMC1221626

[B16] Dixon D. P. Sellars J. D. Kenwright A. M. Steel P. G. (2012). The maize benzoxazinone DIMBOA reacts with glutathione and other thiols to form spirocyclic adducts. *Phytochemistry* 77 171–178. 10.1016/j.phytochem.2012.01.019 22342783

[B17] Ducret A. Trani M. Lortie R. (2006). Comparison between various commercial sources of almond β-glucosidase for the production of alkyl glucosides. *J. Mol. Catal. B Enzym.* 38 91–94. 10.1016/j.molcatb.2005.11.012

[B18] Esen A. (1992). Purification and partial characterization of maize (Zea mays L.) *β-glucosidase*. *Plant Physiol.* 98 174–182. 10.1104/pp.98.1.174 16668611 PMC1080166

[B19] Esen A. Blanchard D. J. (2000). A specific β-glucosidase-aggregating factor is responsible for the β-glucosidase null phenotype in maize. *Plant Physiol.* 122 563–572. 10.1104/pp.122.2.563 10677449 PMC58893

[B20] Esen A. Gungor G. (1993). “Stability and activity of plant and fungal β-glucosidases under denaturing conditions,” in *Proceedings of the ACS Symposium Series. Biochemistry and Molecular Biology* Vol. 533 ed. Esen A. (Washington, DC: American Chemical Society), 214–239. 10.1021/bk-1993-0533.ch015

[B21] Falk A. Taipalensuu J. Ek B. Lenman M. Rask L. (1995). Characterization of rapeseed myrosinase-binding protein. *Planta* 195 387–395. 10.1007/BF00202596 7766044

[B22] Fomunyam R. T. Adegbola A. A. Oke O. L. (1985). The stability of cyanohydrins. *Food Chem.* 17 221–225. 10.1016/0308-8146(85)90072-X

[B23] Fowler T. (1993). “β-glucosidases: Biochemistry and Molecular Biology,” in *Proceedings of the ACS Symposium Series 533*, ed. Esen A. (Washington, DC: American Chemical Society), 56–65.

[B24] Frey M. Chomet P. S. Glawischnig E. Stettner C. Grün S. Winklmair A. (1997). Analysis of a chemical plant defense mechanism in grasses. *Science.* 277 696–699. 10.1126/science.277.5326.696 9235894

[B25] Glauser G. Marti G. Villard N. Doyen G. A. Wolfender J.-L. Turlings T. C. J. (2011). Induction and detoxification of maize 1,4-benzoxazin-3-ones by insect herbivores. *Plant J.* 68 901–911. 10.1111/j.1365-313X.2011.04740.x 21838747

[B26] Grover A. K. Cushley R. J. (1977). Studies on almond emulsin β-D-glucosidase. II. Kinetic evidence for independent glucosidase and galactosidase sites. *Biochim. Biophys. Acta* 482 109–124. 10.1016/0005-2744(77)90359-X 861229

[B27] Halkier B. A. Gershenzon J. (2006). Biology and biochemistry of glucosinolates. *Annu. Rev. Plant Biol.* 57 303–333. 10.1146/annurev.arplant.57.032905.105228 16669764

[B28] He S. M. Withers S. G. (1997). Assignment of sweet almond β-glucosidase as a family 1 glycosidase and identification of its active site nucleophile. *J. Biol. Chem.* 272 24864–24867. 10.1074/jbc.272.40.24864 9312086

[B29] Jeschke V. Gershenzon J. Vassão D. G. (2016a). A mode of action of glucosinolate-derived isothiocyanates: detoxification depletes glutathione and cysteine levels with ramifications on protein metabolism in *Spodoptera littoralis*. *Insect Biochem Mol Biol.* 71 37–48. 10.1016/j.ibmb.2016.02.002 26855197

[B30] Jeschke V. Gershenzon J. Vassão D. G. (2016b). Insect detoxification of glucosinolates and their hydrolysis products. *Adv. Bot. Res.* 80 199–245. 10.1016/bs.abr.2016.06.003

[B31] Kittur F. S. Lalgondar M. Yu H. Y. Bevan D. R. Esen A. (2007). Maize β-glucosidase-aggregating factor is a polyspecific jacalin-related chimeric lectin, and its lectin domain is responsible for β-glucosidase aggregation. *J. Biol. Chem.* 282 7299–7311. 10.1074/jbc.M607417200 17210577

[B32] Kuwar S. S. Pauchet Y. Vogel H. Heckel D. G. (2015). Adaptive regulation of digestive serine proteases in the larval midgut of *Helicoverpa armigera* in response to a plant protease inhibitor. *Insect Biochem. Mol. Biol.* 59 18–29. 10.1016/j.ibmb.2015.01.016 25662099

[B33] Li G.-Z. Vissers J. P. C. Silva J. C. Golick D. Gorenstein M. V. Geromanos S. J. (2009). Database searching and accounting of multiplexed precursor and product ion spectra from the data independent analysis of simple and complex peptide mixtures. *Proteomics* 9 1696–1719. 10.1002/pmic.200800564 19294629

[B34] Maresh J. Zhang J. Lynn D. G. (2006). The innate immunity of maize and the dynamic chemical strategies regulating two-component signal transduction in *Agrobacterium tumefaciens*. *ACS Chem. Biol.* 1 165–175. 10.1021/cb600051w 17163664

[B35] Mattiacci L. Dicke M. Posthumus M. A. (1995). beta-Glucosidase: an elicitor of herbivore-induced plant odor that attracts host-searching parasitic wasps. *Proc. Natl. Acad. Sci. U.S.A.* 92 2036–2040. 10.1073/pnas.92.6.2036 11607516 PMC42418

[B36] Mi L. Di Pasqua A. J. Chung F.-L. (2011). Proteins as binding targets of isothiocyanates in cancer prevention. *Carcinogenesis* 32 1405–1413. 10.1093/carcin/bgr111 21665889 PMC3179418

[B37] Mithöfer A. Boland W. (2012). Plant defense against herbivores: chemical aspects. *Annu. Rev. Plant Biol.* 63 431–450. 10.1146/annurev-arplant-042110-103854 22404468

[B38] Mkpong O. E. Yan H. Chism G. Sayre R. T. (1990). Purification, characterization, and localization of linamarase in cassava. *Plant Physiol.* 93 176–181. 10.1104/pp.93.1.176 16667431 PMC1062485

[B39] Morant A. V. Jørgensen K. Jørgensen C. Paquette S. M. Sánchez-Pérez R. Møller B. L. (2008). β-Glucosidases as detonators of plant chemical defense. *Phytochemistry* 69 1795–1813. 10.1016/j.phytochem.2008.03.006 18472115

[B40] Nakano R. T. Yamada K. Bednarek P. Nishimura M. Hara-Nishimura I. (2014). ER bodies in plants of the Brassicales order: biogenesis and association with innate immunity. *Front. Plant Sci.* 5:73. 10.3389/fpls.2014.00073 24653729 PMC3947992

[B41] Nastruzzi C. Cortesi R. Esposito E. Menegatti E. Leoni O. Iori R. (1996). In vitro cytotoxic activity of some glucosinolate-derived products generated by myrosinase hydrolysis. *J. Agric. Food Chem.* 44 1014–1021. 10.1021/jf9503523 10956152

[B42] Niemeyer H. M. (2009). Hydroxamic acids derived from 2-hydroxy-2H-1,4-benzoxazin-3(4H)-one: Key defense chemicals of cereals. *J. Agric. Food Chem.* 57 1677–1696. 10.1021/jf8034034 19199602

[B43] Pentzold S. Zagrobelny M. Roelsgaard P. S. Møller B. L. Bak S. (2014). The multiple strategies of an insect herbivore to overcome plant cyanogenic glucoside defence. *PLoS One* 9:e91337. 10.1371/journal.pone.0091337 24625698 PMC3953384

[B44] Pihakaski S. Pihakaski K. (1978). Myrosinase in Brassicaceae (Cruciferae).3. Effect of ascorbic-acid on myrosinases from *Sinapis alba* L seedlings. *J. Exp. Bot.* 29 1363–1369. 10.1093/jxb/29.6.1363

[B45] Prieto G. Aloria K. Osinalde N. Fullaondo A. Arizmendi J. M. Matthiesen R. (2012). PAnalyzer: a software tool for protein inference in shotgun proteomics. *BMC Bioinformatics* 13:288. 10.1186/1471-2105-13-288 23126499 PMC3548767

[B46] Ray S. Alves P. C. M. S. Ahmad I. Gaffoor I. Acevedo F. E. Peiffer M. (2016). Turnabout is fair play: herbivory-induced plant chitinases excreted in fall armyworm frass suppress herbivore defenses in maize. *Plant Physiol.* 171 694–706. 10.1104/pp.15.01854 26979328 PMC4854684

[B47] Rotrekl V. Nejedlá E. Kucera I. Abdallah F. Palme K. Brzobohatý B. (1999). The role of cysteine residues in structure and enzyme activity of a maize β-glucosidase. *Eur. J. Biochem.* 266 1056–1065. 10.1046/j.1432-1327.1999.00948.x 10583402

[B48] Schramm K. Vassão D. G. Reichelt M. Gershenzon J. Wittstock U. (2012). Metabolism of glucosinolate-derived isothiocyanates to glutathione conjugates in generalist lepidopteran herbivores. *Insect Biochem. Mol. Biol.* 42 174–182. 10.1016/j.ibmb.2011.12.002 22193392

[B49] Sekhon R. S. Lin H. Childs K. L. Hansey C. N. Buell C. R. de Leon N. (2011). Genome-wide atlas of transcription during maize development. *Plant J.* 66 553–563. 10.1111/j.1365-313X.2011.04527.x 21299659

[B50] Shevchenko A. Tomas H. Havliš J. Olsen J. V. Mann M. (2007). In-gel digestion for mass spectrometric characterization of proteins and proteomes. *Nat. Protoc.* 1 2856–2860. 10.1038/nprot.2006.468 17406544

[B51] Shulman M. L. Shiyan S. D. Khorlin A. Y. (1976). Specific irreversible inhibition of sweet-almond β-glucosidase by some β-glycopyranosylepoxyalkanes and β-D-glucopyranosyl isothiocyanate. *Biochim. Biophys. Acta.* 445 169–181. 10.1016/0005-2744(76)90170-4 8136

[B52] Subramanyam S. Smith D. F. Clemens J. C. Webb M. A. Sardesai N. Williams C. E. (2008). Functional characterization of HFR1, a high-mannose N-glycan-specific wheat lectin induced by Hessian fly larvae. *Plant Physiol.* 147 1412–1426. 10.1104/pp.108.116145 18467454 PMC2442546

[B53] Swain E. Poulton J. E. (1994a). Immunocytochemical localization of prunasin hydrolase and mandelonitrile lyase in stems and leaves of *Prunus serotina*. *Plant Physiol.* 106 1285–1291. 12232409 10.1104/pp.106.4.1285 PMC159666

[B54] Swain E. Poulton J. E. (1994b). Utilization of amygdalin during seedling development of *Prunus serotina*. *Plant Physiol.* 106 437–445. 12232341 10.1104/pp.106.2.437 PMC159548

[B55] Takechi K. Sakamoto W. Utsugi S. Murata M. Motoyoshi F. (1999). Characterization of a flower-specific gene encoding a putative myrosinase binding protein in *Arabidopsis thaliana*. *Plant Cell Physiol.* 40 1287–1296. 10.1093/oxfordjournals.pcp.a029517 10682349

[B56] Takeda M. Sugimori N. Torizawa T. Terauchi T. Ono A. M. Yagi H. (2008). Structure of the putative 32 kDa myrosinase-binding protein from Arabidopsis (At3g16450.1) determined by SAIL-NMR. *FEBS J.* 275 5873–5884. 10.1111/j.1742-4658.2008.06717.x 19021763 PMC2702212

[B57] Terra W. R. Ferreira C. (1994). Insect digestive enzymes: properties, compartmentalization and function. *Comp. Biochem. Physiol. B Biochem. Mol. Biol.* 109 1–62. 10.1016/0305-0491(94)90141-4

[B58] Vic G. Biton J. Le Beller D. Michel J.-M. Thomas D. (1995). Enzymatic glucosylation of hydrophobic alcohols in organic medium by the reverse hydrolysis reaction using almond-β-D-glucosidase. *Biotechnol. Bioeng.* 46 109–116. 10.1002/bit.260460204 18623270

[B59] Winter D. Vinegar B. Nahal H. Ammar R. Wilson G. V. Provart N. J. (2007). An “Electronic Fluorescent Pictograph” browser for exploring and analyzing large-scale biological data sets. *PLoS One* 2:e718. 10.1371/journal.pone.0000718 17684564 PMC1934936

[B60] Wittstock U. Burow M. (2010). Glucosinolate breakdown in Arabidopsis: mechanism, regulation and biological significance. *Arabidopsis Book* 8 e0134. 10.1199/tab.0134 22303260 PMC3244901

[B61] Wouters F. C. Reichelt M. Glauser G. Bauer E. Erb M. Gershenzon J. (2014). Reglucosylation of the benzoxazinoid DIMBOA with inversion of stereochemical configuration is a detoxification strategy in lepidopteran herbivores. *Angew Chem. Int. Ed.* 53 11320–11324. 10.1002/anie.201406643 25196135

[B62] Zhou C. Tokuhisa J. G. Bevan D. R. Esen A. (2012). Properties of β-thioglucoside hydrolases (TGG1 and TGG2) from leaves of *Arabidopsis thaliana*. *Plant Sci.* 19 82–92. 10.1016/j.plantsci.2012.02.004 22682567

[B63] Zouhar J. Nanak E. Brzobohatý B. (1999). Expression, single-step purification, and matrix-assisted refolding of a maize cytokinin glucoside-specific β-glucosidase. *Protocol* 17 153–162. 10.1006/prep.1999.1108 10497081

[B64] Zouhar J. Vévodová J. Marek J. Damborský J. Su X. D. Brzobohatý B. (2001). Insights into the functional architecture of the catalytic center of a maize β-glucosidase Zm-p60.1. *Plant Physiol.* 127 973–985. 10.1104/pp.010712 11706179 PMC129268

