# Supplementary material for: Plant Defensive β-Glucosidases Resist Digestion and Sustain Activity in the Gut of a Lepidopteran Herbivore
Source: Front Plant Sci. 2018 Oct 8;9:1389. doi: 10.3389/fpls.2018.01389 (PMC6186830; doi:10.3389/fpls.2018.01389)
Supplement: TABLE S1 — Maize proteins detected in S. littoralis frass extracts and their predicted functions, number of peptides observed by LC-MS and corresponding sequence coverage, and leaf expression levels (B73 maize). [file Table_1.pdf]

Protein Identifier	Predicted function	# Matched peptides	% Sequence coverage	Expression top leaf (V3)
GRMZM2G064159	Agmatine deiminase	7	20,5	7960,78
GRMZM2G374302	Arginine decarboxylase	13	34,0	549,51
GRMZM2G172204	BGAF1	6	41,8	not found
GRMZM2G453805	Chitinase	4	8,8	1609,16
GRMZM2G016890	Glycosyl hydrolase ZmGlu1	7	15	9805,84
GRMZM2G172369	Glycosyl hydrolase	16	21,9	8638,92
GRMZM2G147687	Glycosyl hydrolase	8	20,1	44620,73
GRMZM2G080839	Oxidoreductase	6	22,0	287,74
GRMZM2G084279	Oxidoreductase	5	31,7	15951,26
GRMZM2G163749	Peptidase	5	11,0	1402,54
GRMZM2G448001	Peptidase	7	18,1	722,19
GRMZM2G031572	Peptidase	6	19,1	11423,93
GRMZM2G126261	Peroxidase	5	28,8	3290,47
GRMZM2G135108	Peroxidase	6	20,0	495,63
GRMZM2G450233	Peroxidase	7	23,0	1583,12
